# Bidirectional rotation control of a carbon fiber in nematic liquid crystal using AC electric field

**DOI:** 10.1038/s41598-020-75644-y

**Published:** 2020-10-29

**Authors:** Jun-Yong Lee, Jeong-Seon Yu, Jong-Hyun Kim

**Affiliations:** 1 Department of Physics, Chungnam National University, 99 Daehak-ro, Yuseong-gu, Daejeon, 34134 Korea; 2 Institute of Quantum Systems, Chungnam National University, Daejeon, Korea

**Keywords:** Nanoscience and technology, Physics, Nanoscale materials, Carbon nanotubes and fullerenes, Graphene, Nanowires, Two-dimensional materials, Materials science, Soft materials, Colloids, Liquid crystals

## Abstract

Colloidal particles dispersed in nematic liquid crystals are aligned along the orientation that minimizes the elastic free energy. Through applying an electric field to a nematic colloidal system, the orientation of the director can change. Consequently, colloidal particles realign to minimize the total free energy, which is the sum of the elastic and electric free energies. Herein, we demonstrate that if the preferred rotation directions given by the electric and elastic free energies are different during realignment, the rotation direction of the particle can be controlled by how we apply the electric field. When the strength of the electric field gradually increases, the particles rotate in the same direction as the rotation of the director. However, when a sufficiently high electric field is suddenly applied, the particles rotate in the opposite direction. In this study, we analyzed the effect of free energy on the bidirectional rotation behavior of the particles using a theoretical model. This study provides an effective approach to control the rotational behavior of colloidal particles over a wide-angle range between two orientational local minima.

## Introduction

Carbon fibers (CFs) are rod-like structured carbon compounds, which are approximately 5–10 $\mathrm{\upmu m}$ in diameter and a few tens of microns in length. CFs consist of graphene-like carbon layers and have excellent electrical and mechanical properties^1^. Graphene is a representative two-dimensional material, which presents anisotropies between in-plane and inter-layer directions^2^. The graphene-like layers of CFs form a concentric cylinder so that CFs exhibit anisotropies between the direction parallel to the fiber axis and the direction perpendicular to the fiber axis. Several studies have been conducted to improve the electrical and mechanical properties of materials such as CFs, by controlling the orientation of their fibers^3–6^.

Liquid crystals (LCs) are highly suitable for studying particles exhibiting anisotropies. Among several types of LCs, nematic liquid crystals (NLCs) are widely investigated, because they show orientational order and fluidity^7,8^. The orientational order implies that the molecular axes of LC molecules are aligned in a preferred direction, called the director. The alignment of the director is determined by the anchoring and elastic properties in LCs^9^. The distortion of the director by external stimuli increases the elastic free energy, and thus makes the system unstable.

The direction of rod-shaped colloidal particles that are dispersed in NLCs can be controlled by modifying the anchoring and elastic properties of NLCs. When colloidal particles are dispersed in NLCs, their surfaces interact with the director. Then, this interaction propagates into the bulk and affects the behavior of the director in the bulk^10–12^. Interactions between the surfaces of the colloidal particles and the director induce a deformation of the director field, and the colloidal particles align along the orientation that minimizes the deformation energy. In other words, particles have a preferred direction in NLCs. Therefore, the orientational behavior of the particles can be induced by controlling the surface condition of the confining cell or exerting external stimuli such as magnetic and electric fields.

Several studies have been conducted to control anisotropic materials such as nano-, micro-rods, and carbon nanotubes (CNTs), using the interactions between the particles and the director^13–21^. For instance, induced local alignment patterns of CNTs using a local electric field in a uniformly aligned LC cell has been reported^22^. Further, another study confirmed that the alignment of rod-shaped particles depends on their surface condition, i.e., when the particles have an axial or homeotropic anchoring condition, they align in the direction parallel or perpendicular to the far-field director, respectively^23^. Therefore, the orientational order of particles can be controlled by changing their surface anchoring conditions. Theoretical approaches have hypothesized the various possibilities of LC and CNT interactions^24,25^. Studies on individual rod-shaped particles had also been reported. The precise static and dynamic properties of individual particles have been investigated by modifying the strength of the electric field^26^. However, the symmetry of these particles and the structure of the cell have not been considered. Moreover, their orientational behavior can be controlled only in the 90° angle range.

In NLCs, there is a 180° symmetry; consequently, the orientation of particles also has a 180° rotational symmetry in the same condition. Hence, they exhibit two local minima. When an AC electric field with a sufficiently high frequency is applied, the electric field also has the same symmetry. However, particles generally rotate only in one direction within less than $90^\circ$; thus, we cannot typically orient them into the two local minima.

In this study, we confirmed that both local minima could be used to obtain an extended range to control the particle rotation. These results may be applied to particles exhibiting symmetry breaking. Herein, we explain the mechanism and the theoretical model of controlled wide-angle rotation using CFs.

## Results

**Figure 1:**
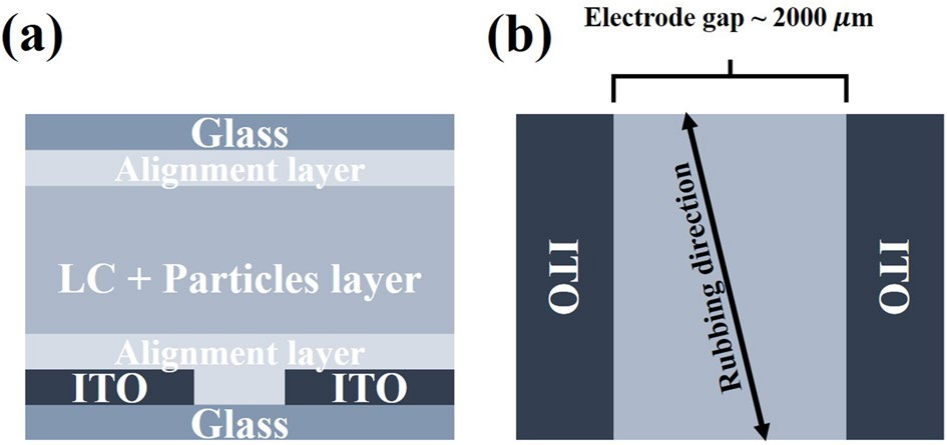
Schematic diagram of the cell used in the experiment. (**a**) and (**b**) are side view and top view, respectively. To clarify the cell structure, we adjusted the relative size of the cell in the diagram. We set the rubbing direction at 10$^\circ$–15$^\circ$ about the axis perpendicular to the electric field.

We prepared a cell, as shown in Fig. 1, and injected an LC + CF mixture into the cell. When the electric field is in the direction perpendicular to the rubbing direction, the rotational direction of the director is randomly determined. To prevent this, we controlled the rubbing direction to be slightly rotated from the perpendicular direction, as shown in Fig. 1b.

We applied an electric field through two different methods. One method was to increase the strength of the electric field gradually. The other was to suddenly apply a sufficiently strong electric field and then gradually decrease the electric field strength. A polarizing optical microscope was used to observe the response of the particles.

When an electric field is applied to particles dispersed in an isotropic fluid, they interact only with the electric field. Consequently, the particles can be oriented in the direction that minimizes the electric free energy. In CFs, we can define the orientation of CFs as fiber axis. In contrast, for particles with irregular shapes such as black phosphorus (BP) or graphene flakes, defining a new directional axis along the electric field is required because they present no noticeable reference axis. Hence, we had defined the electric axis as the orientation parallel to the applied electric field. In the nematic phase, it is difficult to determine the electric axis because particles interact not only with the electric field but also with the director. Therefore, we determined the electric axis at a temperature above the nematic-isotropic transition temperature ${\mathrm{T}}_{\mathrm{NI}}$.

Because we applied an AC field with a higher frequency than the response of the director, the anode and cathode were not differentiated. Hence, the electric field had a rotational symmetry of 180°. The electric free energy corresponded to the same rotational symmetry, thus indicating that the particles had two stable orientations as a response to the electric field. However, particles rotate under an applied electric field along the direction in which the initial angle between the electric field and the electric axis is less than $90^\circ$. Therefore, it is difficult to confirm both stable orientations in isotropic fluids. In contrast, in a nematic colloidal system, the rotational direction of the particle is determined not only by the electric field but also by both elastic deformation and anchoring energy. Using the properties of the nematic colloidal system, we derived results that could not be obtained from testing in the isotropic fluid.

**Figure 2:**
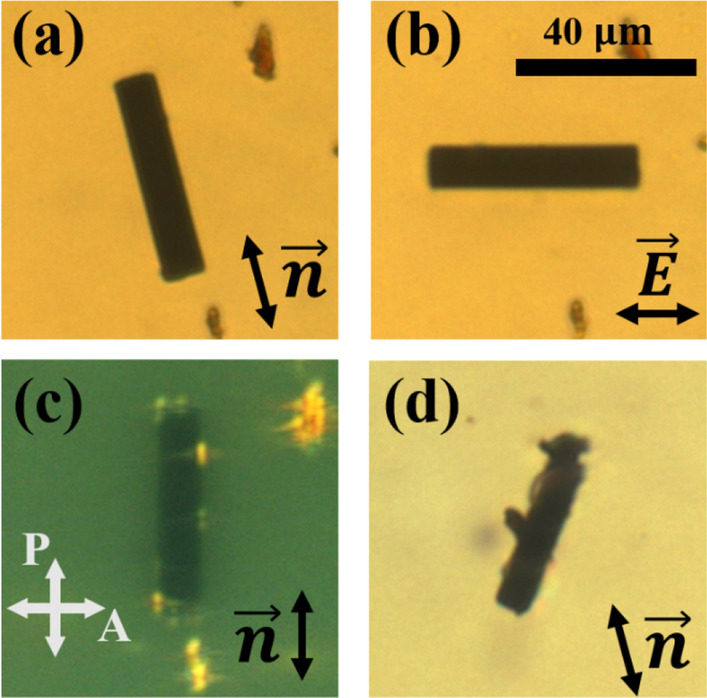
(**a**) Optical microscope image of the symmetric CF at the ground state. It is aligned parallel to the rubbing direction in the nematic phase. (**b**) Image of CF when an electric field of 0.080 V/$\mathrm{\upmu m}$ was applied. LC is in the isotropic phase. The CF is aligned parallel to the electric field. (**c**) Image of the CF observed in crossed polarizers through polarizing optical microscope. To obtain the image when it is dark, the rubbing and director directions coincide with the polarizer direction. We modified brightness and contrast to clarify the image. Although small dust particles were attached to the CFs, it seems that they hardly affect the response of the CF (**d**) The image of the irregular shaped CF. It is not aligned parallel to the rubbing and director direction.

Colloidal particles dispersed in NLCs have a stable orientation because of their surface condition and shape. In the absence of an electric field externally applied, for symmetric CFs, the fiber axis is aligned parallel to the rubbing direction as shown in Fig. 2a. Because the electric axis is the same as the fiber axis, the fiber axis is parallel to the applied electric field in the isotropic phase (Fig. 2b). As observed in the image from the polarizing optical microscope, there was little deformation around CFs; thus, we considered that the director is parallel to the fiber axis of CFs (Fig. 2c). However, irregularly shaped CFs were not aligned parallel to the rubbing direction, even in the absence of an electric field (Fig. 2d).

When a CF is placed in an LC cell with a uniformly aligned director, it induces deformation of the director due to the interactions at surface of the CF with the director. This deformation increases the elastic free energy. The elastic free energy can be expressed as a combination of splay, twist, and bend deformation. The elastic constants of each deformation component are ${K}_{1}, {K}_{2}, \mathrm{and}\ {K}_{3},$ equal to 6.5, 3.5, and 9.8 pN, respectively^27^. The elastic free energy density (*f*) can be obtained as follows^28^:

1 $$f=\frac{1}{2}\left[{K}_{1}{\left(\nabla \cdot \overset{\lower0.5em\hbox{$\smash{\scriptscriptstyle\rightharpoonup}$}} {n} \right)}^{2}+{K}_{2}{\left( \overset{\lower0.5em\hbox{$\smash{\scriptscriptstyle\rightharpoonup}$}} {n} \cdot \nabla \times \overset{\lower0.5em\hbox{$\smash{\scriptscriptstyle\rightharpoonup}$}} {n} \right)}^{2}+{K}_{3}{\left( \overset{\lower0.5em\hbox{$\smash{\scriptscriptstyle\rightharpoonup}$}} {n} \times \nabla \times \overset{\lower0.5em\hbox{$\smash{\scriptscriptstyle\rightharpoonup}$}} {n} \right)}^{2}\right]$$

where $$\overset{\lower0.5em\hbox{$\smash{\scriptscriptstyle\rightharpoonup}$}} {n}$$ is the director vector. This equation can be simplified using a *K* constant as follows:

2 $$f=\frac{1}{2}K\left[{\left(\nabla \cdot \overset{\lower0.5em\hbox{$\smash{\scriptscriptstyle\rightharpoonup}$}} {n} \right)}^{2}+{\left(\nabla \times \overset{\lower0.5em\hbox{$\smash{\scriptscriptstyle\rightharpoonup}$}} {n} \right)}^{2}\right]$$

where $K$ (~ 6.6 pN) is the average value of the three elastic constants.

**Figure 3:**
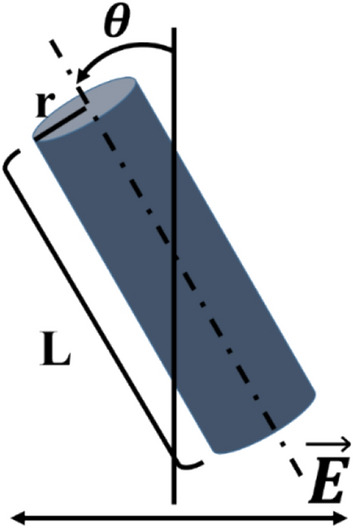
Schematic diagram indicating the angle and physical parameters, $\theta$ is the angle between the fiber axis, and the direction perpendicular to the electric field.

Considering a cylindrical CF dispersed in a uniformly oriented LC cell, the director maintains an alignment parallel to the fiber axis at the surface of the CF due to strong anchoring strength. Because the length of the CF is finite, the director can be deformed around a limited region. The deformation range is proportional to the fiber radius, and the deformation energy density is inversely proportional to the square of that radius. In contrast, the deformation volume is proportional to the square of the radius. Because the deformation energy is the sum of the deformation energy density within the volume, the deformation energy is not dependent on the radius of the fiber. From these assumptions, we can express the elastic deformation energy (${F}_{deform}$) as follows^29^:

3 $${F}_{deform}=\frac{\pi KL}{\mathrm{ln}(a/2) }{(\theta -{\theta }_{rub})}^{2}$$

where ${\theta }_{rub}$ is the rubbing direction and $\theta$ is the angle of the fiber axis; *a* is defined as *L/r* where *L* is the length and *r* is the radius (Fig. 3). According to Eq. (3), if no electric field is applied, symmetric CFs in uniformly oriented LC cells align in the direction parallel to the rubbing direction, and the deformation is proportional to square of deviation angle. This was expected because of the surface condition and the axial symmetry of the CFs. However, the orientation of CFs may change if they exhibit an irregular shape with symmetry breaking. In such particular cases, we can slightly modify Eq. (3) as

4 $${F}_{deform}=\frac{\pi KL}{\mathrm{ln}(a/2) }{(\theta -{\theta }_{rub}-{\theta }_{0})}^{2}$$

where ${\theta }_{0}$ is the angle between the rubbing direction and the CF axis when the fiber has an irregular shape in the absence of an electric field. This is due to symmetry (or surface alignment) breaking of the CF. ${\theta }_{0}$ increases as the CF is further apart from its cylindrical shape. If CFs have weak anchoring strength, we should consider the director-orientation-dependent anchoring energy. However, we may neglect the anchoring energy assuming that CFs exhibit a strong anchoring strength.

When an electric field is applied to the system, the director and CFs exhibit a response; thus, a change in the total free energy occurs. The electric free energy density (${f}_{elec}$) that originates from the interaction between the electric field and the director can be expressed as

5 $${f}_{elec}=-\frac{1}{2}{\epsilon }_{0}\Delta {\epsilon }_{LC}{E}^{2}{\mathrm{sin}}^{2}\phi$$

where ${\epsilon }_{0}$ is the electric permittivity in vacuum, $\Delta {\epsilon }_{LC}\left(\sim 12\right)$ is the dielectric anisotropy of 5CB, and $\phi$ is the director angle with respect to the axis perpendicular to the applied electric field. Considering the CF as a conducting cylinder with a particular length (*L*) and radius (*r*), it presents a dipole moment in the direction parallel to the fiber axis. The dipole moment can be obtained by the following expression^30^:

6 $$p={C}_{1}{C}_{2}\frac{4\uppi {\upepsilon }_{0}\kappa {L}^{3}}{24\left[lna-1\right]}\left[1+\frac{\frac{4}{3}-ln2}{lna-1}\right]\cdot E$$

where ${C}_{1}$ (~ 2/5) is a coefficient used to correct deviations from a perfect electrical conductor. Because Eq. (6) was derived from the assumption that CF has a dipole moment in the direction parallel to the fiber axis considering a perfect cylindrical shape, we have to correct this equation to make it valid in cases where CF is not a perfect cylindrical particle. The coefficient ${C}_{2}$ (~ 1/6) is a correction to such geometrical and electric conditions due to symmetry breaking. We may define ${C}_{2}$ as the value that reflects the behavior of CF as expressed in Eq. (6). $\kappa \left(\sim 10\right)$ is the relative dielectric constant of the medium surrounding the dispersed CF. LCs exhibit anisotropy of the relative dielectric constant; therefore, we used an average value. Because the two axes are affected by ${\upepsilon }_{\perp } \left(\sim 6\right)$ but only one axis is affected by ${\upepsilon }_{\parallel } \left(\sim 18\right)$, we calculated the average value as $({\upepsilon }_{\perp }+2{\epsilon }_{\parallel })/$3^31^. From these assumptions, the electric free energy $({F}_{{CF}_{elec}})$ under an applied electric field is calculated as

7 $${F}_{{CF}_{elec}}=-{C}_{1}{C}_{2}\frac{1}{2}\frac{4\pi {\epsilon }_{0}\kappa {L}^{3}}{24\left[lna-1\right]}\left[1+\frac{\frac{4}{3}-ln2}{lna-1}\right]\cdot {\mathrm{E}}^{2}{\mathrm{sin}}^{2}\theta$$

By applying the proposed equations, we can predict the response of CFs under an applied electric field, and validate it against experimental results.

When an electric field is applied to the LC + CF system, the director and CFs undergo rotation to minimize the total free energy as a response to the electric field. Herein, we should consider not only the effect of the electric field but also the simultaneous interaction between the director and CFs. However, this may require a highly complex calculation. Thus, we simplified it assuming that the director around a CF is affected only by the interaction between the director and the electric field, irrespective of the interaction between the director and the CF. Because the volume of CFs is negligible compared to that of the LC, the effect of CFs on the response of the director is negligible. Therefore, we can express the orientation of the director as a function of the electric field (${\theta }_{LC}\left(E\right))$. From Eqs. (2) and (5), the response of the director assuming that only the interaction between the director and the electric field exists can be expressed as

8 $${f}_{director}=K{\left[\frac{\left(\phi -{\theta }_{rub}\right)}{d/2}\right]}^{2}-\frac{1}{2}{\upepsilon }_{0}\mathrm{\Delta \epsilon }{\mathrm{E}}^{2}{\mathrm{sin}}^{2}\phi$$

where $${f}_{director}$$ denotes the free energy density in the LC under an applied electric field and *d* is the cell thickness. We can determine the $${\theta }_{LC}(E)$$ as the value minimizes Eq. (8) for a particular electric field strength. Assuming that the director is not affected by interactions between the director and CFs, we can express the free energy of the CF ($${F}_{CF})$$ using the following equation:

9 $${F}_{CF}=\frac{\pi KL}{\mathrm{ln}(a/2) }{(\theta -{\theta }_{LC}-{\theta }_{0})}^{2}-{C}_{1}{C}_{2}\frac{4\pi {\epsilon }_{0}\kappa {L}^{3}}{24\left[lna-1\right]}\left[1+\frac{\frac{4}{3}-ln2}{lna-1}\right]\cdot {\mathrm{E}}^{2}{\mathrm{sin}}^{2}\theta$$

${\theta }_{LC}$ becomes constant at a particular electric field strength, considering Eq. (8). Hence, the CF orientation ($\theta$) can be determined by minimizing Eq. (9).

**Figure 4:**
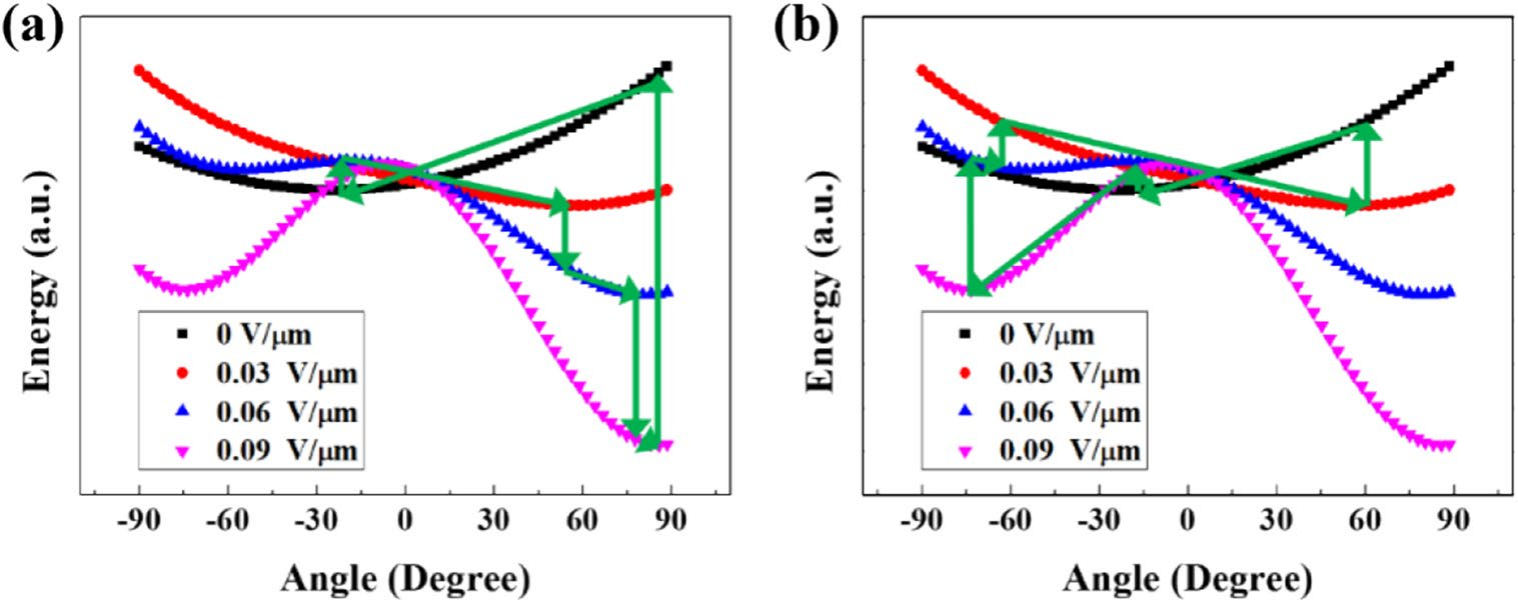
Schematic diagram explaining the bidirectional rotation of CFs in the experiment. The curves are the plots of ${F}_{CF}$ from Eq. (9) by the strength of an electric field. The CF was stable at the local minima of each curve. (**a**) When the electric field was gradually increased from 0 V/$\mathrm{\upmu m}$. (**b**) When a strong electric field of 0.09 V/$\mathrm{\upmu m}$ was applied and then decreased steadily. In both images, the processes follow the arrows starting from the minimum of the black curve (0 V/$\mathrm{\upmu m}$).

We plotted Eq. (9) by varying the electric field strength values, ***E*** (Fig. 4). Only one local minimum was observed under a low electric field. With increasing electric field, the shape of the ${F}_{CF}$ curve changed. When the electric field was higher and its strength exceeded a specific value, another local minimum appeared. A CF can be oriented along the local minimum of ${F}_{CF}$ at a particular electric field strength value. In this process, the CF does not always move to the global minimum, but moves toward the orientation easier to move.

For instance, when no electric field was applied, the CF located near − 25$^\circ$ (the lowest location of the black line in Fig. 4). However, at an applied electric field of 0.03 V/$\mathrm{\upmu m}$, the local minimum differed from − 25$^\circ$ and the CF angle was expected to change to near 60$^\circ$ (as shown by the arrows in Fig. 4a). In contrast, when the applied electric field suddenly changed from 0 to 0.09 V$/\mathrm{\upmu m}$, the shape of the total free energy curve against the angle changed as denoted by the purple line in Fig. 4. Consequently, two local minima occurred near − 70$^\circ$ and 80$^\circ$. The right-side minimum is the global minimum; nevertheless, the CF angle drifted to the left-side minimum because the CF had to pass an energy barrier to move to the right-side minimum.

Additionally, when the electric field strength increased from 0 to 0.05 V/$\mathrm{\upmu m}$, the total free energy decreased to approximately ${10}^{-15}\; \mathrm{ J}$, as shown in Fig. 4. Because this value is larger than that of the thermal energy scale (~ ${10}^{-21}\; \mathrm{J}$), we concluded that thermal fluctuation did not affect the motion of the particle.

**Figure 5:**
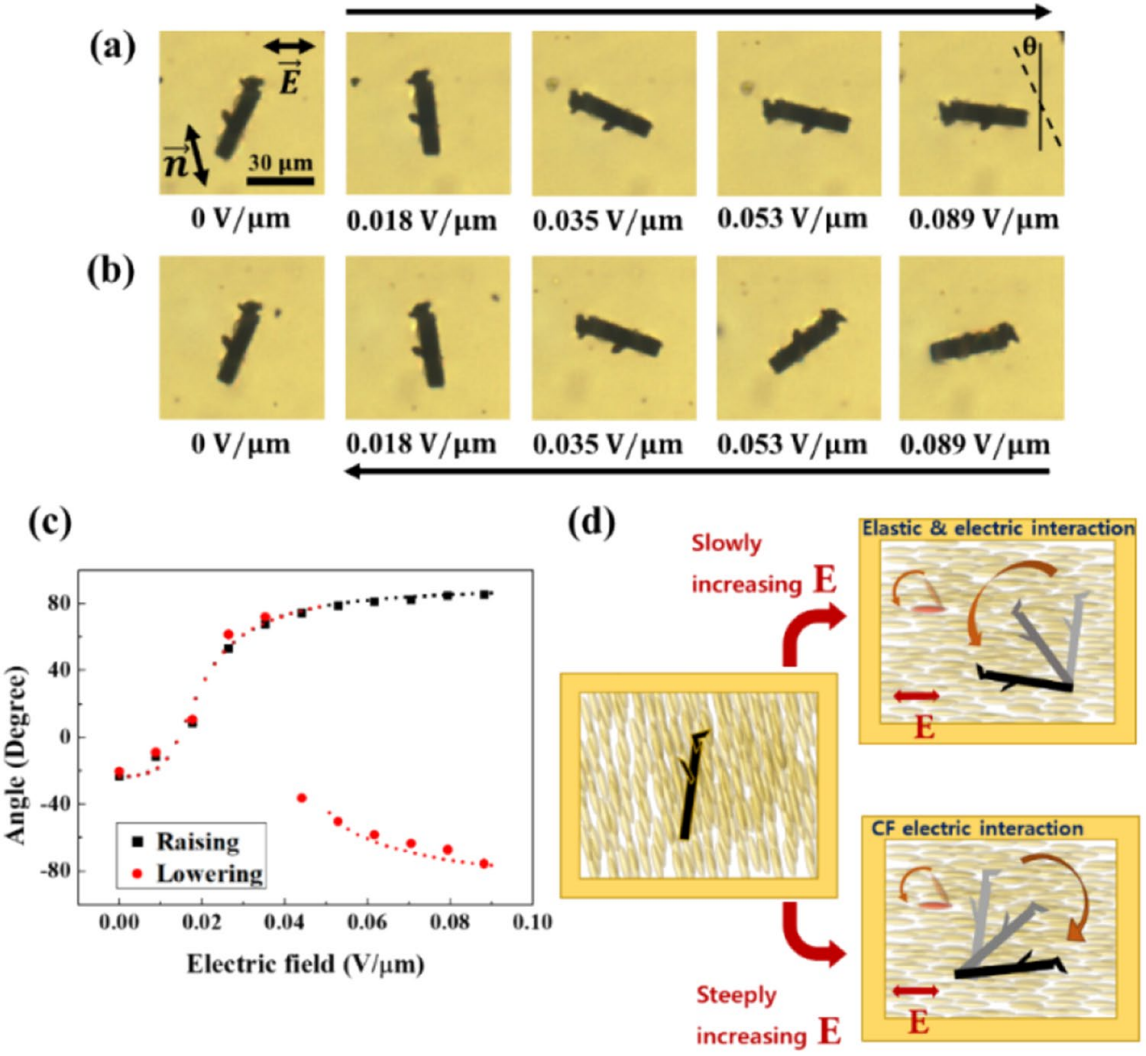
(**a**) Images of the CF with a gradual increase in the strength of the electric field. (**b**) The images of the CF when a strong electric field was suddenly applied, followed by a steady decrease. The experiment proceeded from the right image to the left image. (**c**) The graph of angle by an electric field for the results of (**a**) and (**b**). As shown in the rightmost image of (**a**), we defined the angle as the degree of counterclockwise rotation from the vertical axis. The large and small marks indicate the experimental and calculation results, respectively. The long arrows above (**a**) and under (**b**) indicate the order in which the experiment proceeds. (**d**) Schematic diagram of CF rotation in the experiments.

We investigated the response of the irregularly shaped CF (Fig. 2) in the LC + CF cell through two different methods to apply the electric field defined herein; i.e., the first one by increasing the strength gradually and the second one suddenly applying a strong electric field and then decreasing it (Fig. 5). Through the first method (gradually increasing electric field strength), the CF rotated counterclockwise steadily (Fig. 5a). In contrast, from the second method, we observed that CF exhibited significant clockwise rotation under a strong electric field, and rotated counterclockwise as the electric field strength decreased. The counterclockwise rotation was observed until the electric field reached the specific strength where two local minima occurred, at approximately 0.04 V/$\mathrm{\upmu m}$. Finally, when the electric field decreased, the CF rotated clockwise again until it was in the ground state (Fig. 5b). We analyzed these processes using the angle definition in Fig. 3 by plotting the values. The experimental results were compared with the calculated results from Eq. (9) (Fig. 5c); it can be observed that similar values were obtained.

In addition to the interpretation in terms of the free energy, we explained the bidirectional orientation behavior of CF using the competition between interactions with the electric field and the director field. When the CF was in the ground state (Fig. 5a), the director exhibited a tendency to rotate counterclockwise under an applied electric field. The interaction between the director and the CF contributed to the counterclockwise rotation of the CF. In contrast, direct interaction between the electric field and the CF contributed to clockwise rotation until the angle of CF reached 0$^\circ$. Therefore, the rotation direction of the CF in the ground state was dependent on the dominant interaction. When the electric field strength increased gradually, the director rotated even under a weak electric field, and contributed to the CF rotation owing to the elastic deformation energy. The weak electric field had no significant contribution to the CF rotation. In this case, the CF interaction with the director was the dominant; hence, the CF exhibited counterclockwise rotation. Under a suddenly induced strong electric field, the electric field determined the CF rotation even though the director simultaneously rotated. Hence, the CF rotated clockwise (Fig. 5d). This could be confirmed by analyzing the dynamics of the CF.

**Figure 6:**
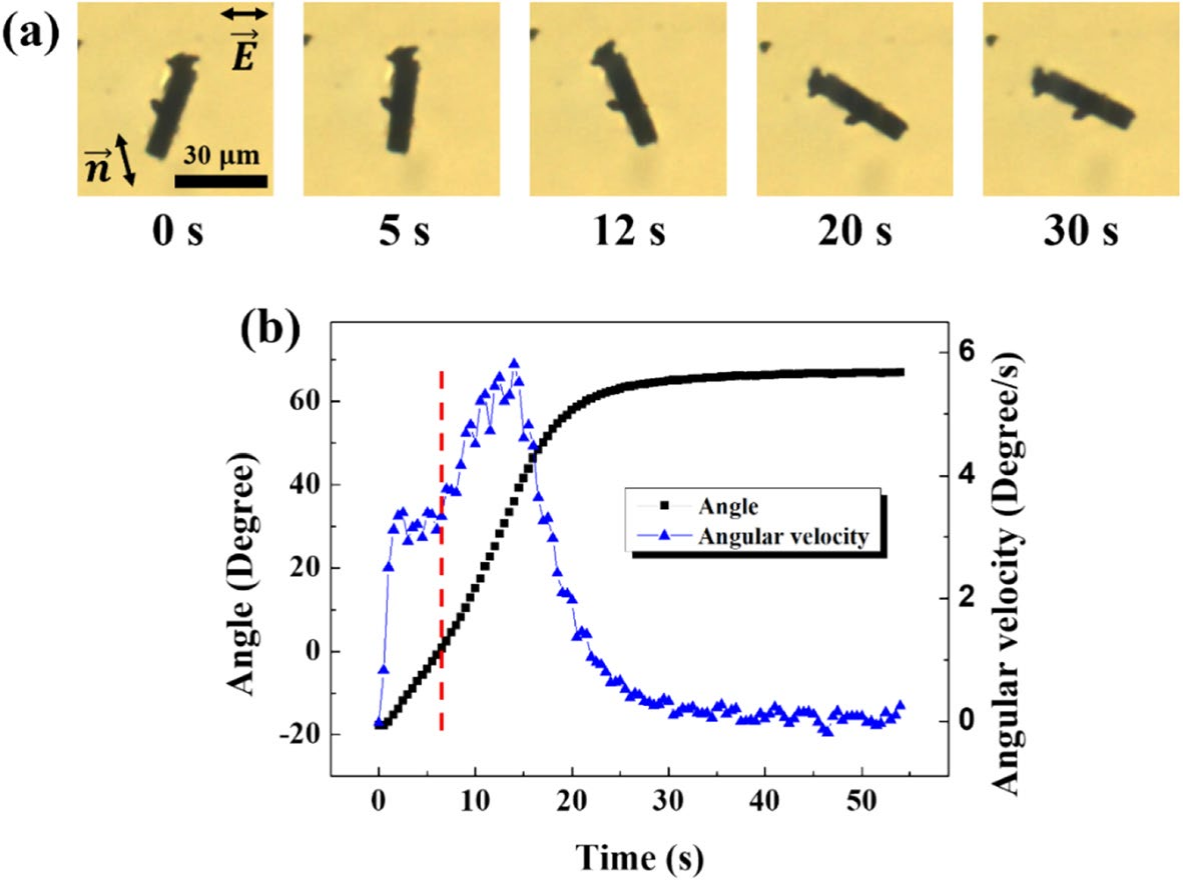
Time-dependent rotational angle variation of CF with decreasing electric field. First, we applied an electric field of 0.045 V/$\mathrm{\upmu m}$ and waited for the CF to reach equilibrium. Next, we changed the strength of the electric field to 0.035 V/$\mathrm{\upmu m}$. (**a**) Images with time lapse. 0 s corresponds to the moment the electric field strength was changed. (**b**) The angle and the angular velocity with time. The red dotted line indicates the moment at which CF reaches 0°.

As shown in Fig. 5c, when the electric field strength decreased to 0.045 V/$\mathrm{\upmu m}$ under a strong electric field, the CF rotated clockwise around the vertical axis. When the electric field strength further decreased to 0.035 V/$\mathrm{\upmu m}$, the CF rotated counterclockwise and passed over the vertical axis, as observed from the images of the process that were captured every 0.5 s (Fig. 6a). We analyzed these images in terms of both timely angle and angular velocity measurements (Fig. 6b). As shown in Fig. 6b, the angular velocity (initially zero), increased rapidly and maintained at a constant value. The rapid increase in angular velocity can be explained by the accelerated rotation due to the elastic deformation effect when the electric field started being decreased. Next, the angular velocity maintained until crossing the red dotted line probably due to a balance among the electric field, elastic deformation, and viscous torque. Here, the interaction with the director contributed to the CF counterclockwise rotation, and the interaction with the electric field contributed to the CF clockwise rotation. Hence, the two effects balanced each other and the angular velocity maintained at a relatively small value. After the CF exceeded the angle of the red dotted line, the effects due to CF interactions with both the electric field and the director contributed to rotation in the same direction, and thus the angular velocity drastically increased and then decayed when a stable state was achieved. This analysis provides an understanding of the rotation behavior of the CF.

## Discussion

The CF particles used in this experiment are denser than 5CB; therefore, they might have dispersed at the bottom of the cell rather than in the middle. However, to simplify the analysis of the results, we assumed that CFs were dispersed in the middle of the cell.

When an electric field is applied, the rotations of the director and the particles require time to be accomplished. Therefore, it would be necessary to consider that time. However, we assumed that the director rotated as soon as the electric field was applied because the rotation of the director is typically much faster than that of the particles; thus, only the rotation time for the particles was considered.

Herein, we calculated the deformation energy using the expression: ${F}_{deform}=\left(\pi KL/\mathrm{ln}(a/2)\right) {\theta }^{2}$ considering the CF has an angle ($\theta$) with respect to the far-field director. Because a perfect cylindrical particle was assumed in this equation, some differences in the case of imperfect cylindrical particles can exist. Nevertheless, the proportionality of the deformation energy to ${\theta }^{2}$ will not change and only the coefficient in the equation may slightly differ.

**Figure 7:**
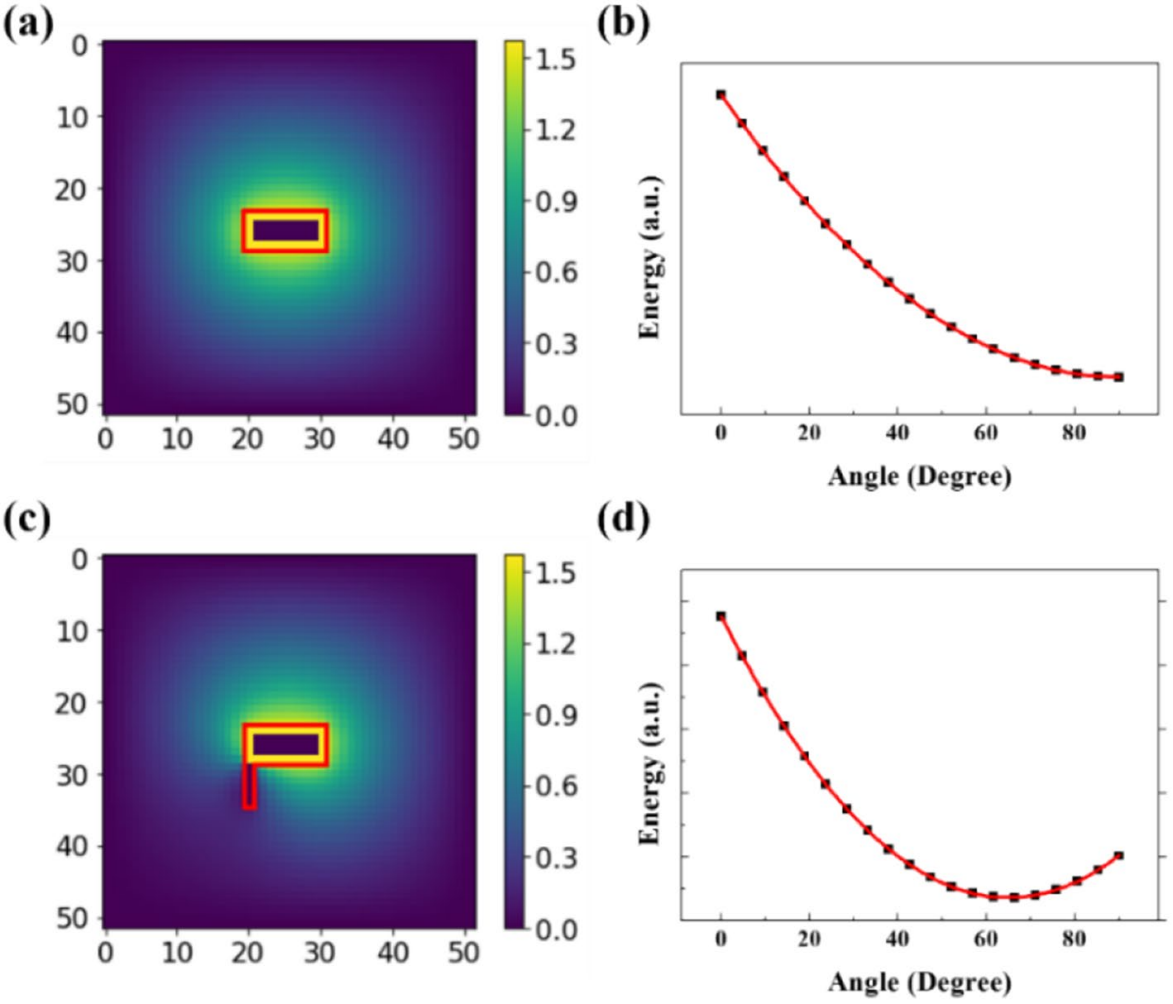
We drew the director distribution around (**a**) the symmetric CF and (**c**) the irregular shaped CF when they were aligned perpendicular to the far-field director. Red boxes in (**a**) and (**c**) indicate the shapes of CFs. In (**c**), we exaggeratedly expressed the irregular part. We plotted the energy by angle for (**b**) the symmetric CF and (**d**) the irregularly shaped CF. The red lines in (**b**) and (**d**) indicate the results of fitting by the equation, $\mathrm{E}=\mathrm{A}{\left(\uptheta -{\uptheta }_{0}\right)}^{\mathrm{P}}+{\mathrm{E}}_{0}$. Comparing this equation with Eq. (3), A is the coefficient of the cylindrical shape of the particle. The value of A was smaller in the symmetric CF than in the irregularly shaped CF. ${\mathrm{E}}_{0}$ is the energy at the ground state. P is the power, which indicates how the energy changes with deformation. In both cases, P was 2. In other words, the energy is proportional to the square of the angle for both cases.

To provide a plausible explanation, we proposed a simple two-dimensional model and numerically estimated the free energy. For the symmetric CF particles, we considered that the CF was rectangular and flat, and the director was aligned parallel to the CF axis at its surface (Fig. 7a). Then, we calculated the most stable orientation for the CF rotation by changing the boundary condition, as illustrated by the outer square in Fig. 7a. The results indicate that the analyzed system is stable when the CF orientation is the same as that of the far-field director and the free energy is proportional to ${\theta }^{2}$ (Fig. 7b). For the irregularly shaped CF particles, we considered the exaggerated model, in which the CF has a tail, and the director is aligned in the direction perpendicular to the CF axis at the tail (Fig. 7c). In this case, the energy is proportional to ${\theta }^{2}$, as in the earlier mentioned case (director aligned parallel to the CF axis), even if the stable angle changed as shown in Fig. 7d. Although this is a simplified model, we confirmed that we could apply Eq. (4) not only to symmetric CFs but also to irregularly shaped CFs. Additionally, we should consider the influence of possible defects. The defects may have a quadrupole nature due to planar anchoring of the CF; thus, it was concluded that defects could rarely affect the symmetry of the CF. Hence, we neglected the influence of defects.

In addition, we assumed that the orientation of the director could be determined only by the electric field strength. In principle, the interaction between the director and the CF should also be considered. Although this assumption differs from the actual interactions, the calculations obtained through the proposed model were in well agreement with the experimental results.

**Figure 8:**
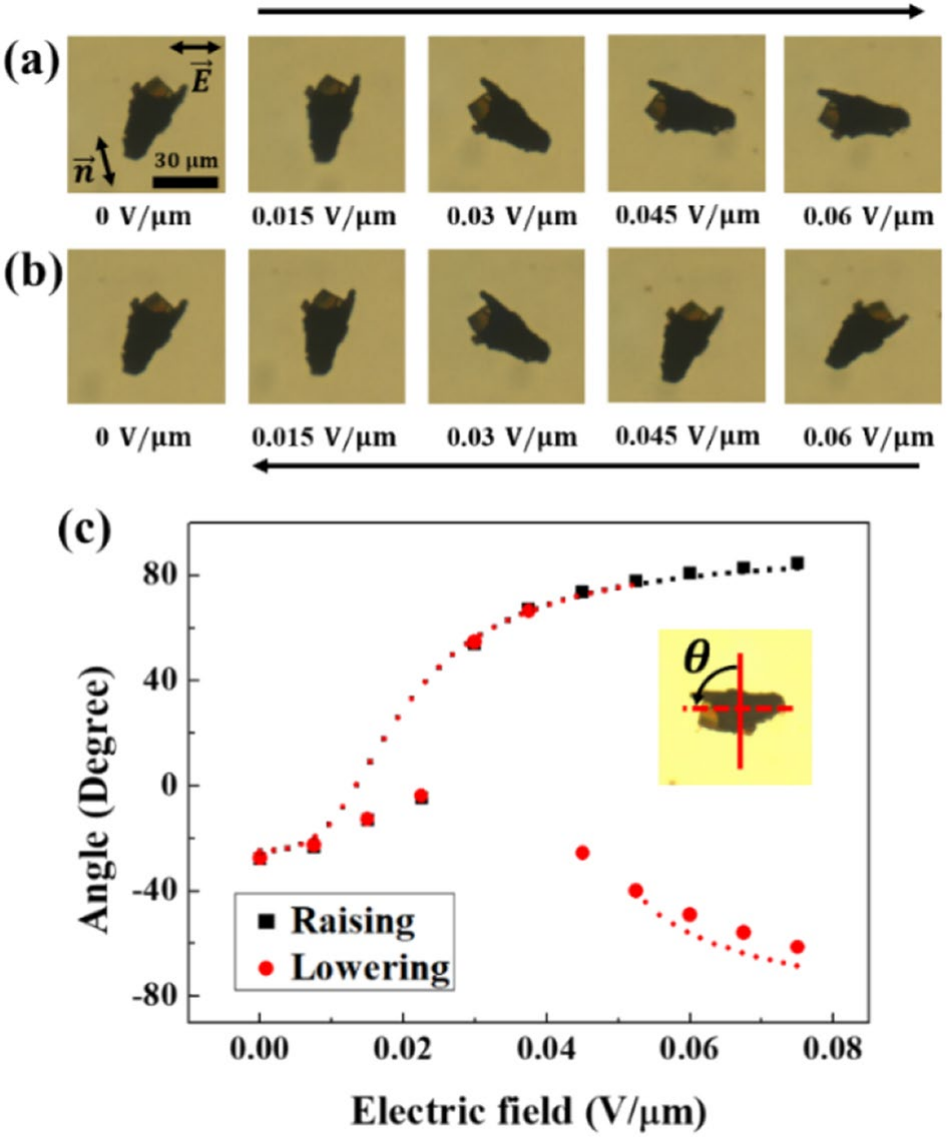
(**a**) Images of BP with a gradual increase in the strength of the electric field. (**b**) Images of BP with decreasing strength of the electric field after suddenly applying a strong electric field. The experiment proceeded from the right image to the left image. (**c**) We analyzed the above images as angle by electric field. The inset is the image of BP when an electric field is applied in the isotropic phase. The large and small marks indicate the experimental and calculation results, respectively. The long arrows above (**a**) and under (**b**) indicate the order in which the experiment proceeds.

We also conducted the same experiment on irregularly shaped two-dimensional materials such as BP and graphene. We observed that they exhibited a behavior similar to that of the CFs. When the electric field strength gradually increased, the particles rotated along with the director. In this experiment, the director rotated counterclockwise. In contrast, the particle rotated in the clockwise direction when a strong electric field was applied and then slowly decreased (Fig. 8a,b). We defined the angle of the particle as that between the electric and vertical axes. However, the electric axis arising from the irregular shapes of the particle was required. Because the deformation effect of nematic phase disappeared in the isotropic phase, the electric axis perfectly aligned with the electric field direction. Hence, we easily determined the electric axis of BP (inset of Fig. 8c).

Due to the highly arbitrary shape of BP, there was a lack of information to explain this behavior. Hence, we assumed that the elastic deformation energy and the electric free energy might be expressed in a simplified form without considering the shape of the particles. The elastic deformation energy in this model was expressed as follows:

10 $${F}_{deform}=K{V}_{deform}{\left(\frac{\theta -{\theta }_{LC}-{\theta }_{0}}{d/2}\right)}^{2}$$

where $\theta$ is the angle with respect to the electric axis perpendicular to the electric field, *d* is the cell thickness, ${V}_{deform}$ is the deformation region, and ${\theta }_{LC}$ and ${\theta }_{0}$ are defined in Eq. (9). Therefore, the electric free energy could be obtained by the following expression:

11 $${F}_{elec}=-\frac{1}{2}{\epsilon }_{0}\Delta {\epsilon }_{particle}{E}^{2}{\mathrm{sin}}^{2}\theta {V}_{particle}$$

where $\Delta {\epsilon }_{particle}$ is the dielectric anisotropy and ${V}_{particle}$ is the volume of the particle. To achieve precise calculations, the dipole moment and the electric free energy considering the shape of the particles should be included. However, we assumed that BP was the material that exhibited anisotropy of dielectric constant. We compared the experimental results with calculations obtained from Eqs. (10) and (11) (Fig. 7c). When analyzing the results, arbitrary values to fit calculations with the experimental results were used. The assumed values were as follows: anisotropy of the particle, $\Delta {\epsilon }_{particle},$ was 50 and the particle size was $30\times 10\times 0.7\; \upmu {\rm m}^{3}\ (\mathrm{L}\times \mathrm{W}\times \mathrm{D})$. The assumed dielectric anisotropy was high. It could be explained with the fact that electrical properties of BP get close to that of conductors as the thickness of BP increases^32,33^.

Some neglected issues are related to actual physical parameters. There was no detailed consideration for the shape of the particle; it was rather assumed that the particles had a rectangular shape. ${V}_{deform}$ was considered as the sum of two quadrangular pyramids above and below the surface of the particle, assuming that the distortion range of the director decreased with increasing distance from the surface of the particle. Because it is difficult to make an appropriate estimation of $\Delta {\epsilon }_{particle}$, we used an arbitrary value that fitted well with the experimental results. The thickness of two-dimensional materials dispersed in the LC cell was also difficult to determine. When this thickness is a few tens of nm, the transmission is sufficiently high and, consequently, the particles show a bright color. However, the dielectric anisotropy is quite high when the particle is very thick and the rotation induced by the electric field is the dominant, which induces the particle to rotate along one direction regardless how the electric field is applied. Hence, we used that arbitrary value to obtain a correct fitting of the experimental results. Compared to the analysis using CFs, we used a highly simplified model and values for irregularly shaped particles. Nevertheless, the assumptions made and the corresponding calculations explained the trend in the experimental results.

## Conclusions

Anisotropic particles dispersed in NLCs are aligned in a particular orientation because of their interaction with the director. When the director rotates because of the effect of an applied electric field, the particles typically rotate in the direction of the director orientation. The rotation behavior of the CF depends on how the electric field is applied when the preferred rotation directions caused by interactions with the director and the electric field are different. If the electric field is increased gradually, the particle rotates following the director rotation. However, in case a strong electric field is suddenly applied, the particle rotates in the direction opposite to the director because the interaction with the electric field is the dominant. We confirmed this trend from experimental results and through calculations using a theoretical model.

When a sufficiently strong AC electric field is applied, the system has two local minima because of the 180° symmetry. In this study, we confirmed both local minima by symmetry breaking of the cell and particles, which has not been experimentally confirmed in the literature. The particles rotated clockwise and counterclockwise in the 180° range. Furthermore, we controlled the orientation of the particles over a wide-angle range.

## Methods

An LC cell sandwiched in two glass sheets was used for the experiments. One sheet was a bare glass substrate, and the other one was an ITO glass substrate containing the electrode pattern for an in-planar electric field. The gap between the electrodes was approximately 2000 $\mathrm{\upmu m}$. Both substrates were spin-coated with a planar aligned polyimide, PIA-PI114-01X (obtained from JNC), and were baked. The substrates were fixed on the cell using a double-sided tape, which resulted in a cell gap of 70 $\mathrm{\upmu m}$. Then, the LC + particle mixture was injected into the cell (Fig. 1). Rubbing of the substrates in a controlled orientation was conducted.

We used 4-cyano-4′-pentylbiphenyl (5CB), which exhibits the nematic phase at the range of $24^\circ \mathrm{C}-36^\circ \mathrm{C}$. The CFs (obtained from Nanoshel) were a few tens microns in length, with a radius of 4 $\mathrm{\upmu m}$ and a density of 2 g/${\mathrm{cm}}^{3}$. We prepared the mixture by adding a small amount of CFs into 5CB and stirring for several minutes using a vortex mixer. A 5-kHz square wave was applied using a function generator and an amplifier.
